# *Paenibacillus plantiphilus* sp. nov. from the plant environment of *Zea mays*

**DOI:** 10.1007/s10482-023-01852-x

**Published:** 2023-06-20

**Authors:** Peter Kämpfer, André Lipski, Lucie Lamothe, Dominique Clermont, Alexis Criscuolo, John A. McInroy, Stefanie P. Glaeser

**Affiliations:** 1 Institut für Angewandte Mikrobiologie, Justus-Liebig-Universität Giessen, Heinrich-Buff-Ring 26–32, 35392 Giessen, Germany; 2 Institut für Ernährungs– und Lebensmittelwissenschaften, Lebensmittelmikrobiologie und –hygiene, Rheinische Friedrich-Wilhelms-Universität Bonn, Bonn, Germany; 3 CNRS, IFB-Core, UMS 3601, Institut Français de Bioinformatique, Evry, France; 4 Institut Pasteur, Bioinformatics and Biostatistics Hub, Université de Paris, 75015 Paris, France; 5 Institut Pasteur, CIP - Collection of Institut Pasteur, Université de Paris, 75015 Paris, France; 6 Department of Entomology and Plant Pathology, Auburn University, Alabama, USA

**Keywords:** Endophyte, *Paenibacillus plantiphilus*, Plant associated, Taxonomy, *Zea mays*

## Abstract

A Gram-strain positive, aerobic, endospore-forming bacterial strain (JJ-246^T^) was isolated from the rhizosphere of *Zea mays*. The 16S rRNA gene sequence similarity comparisons showed a most closely relationship to *Paenibacillus oenotherae* DT7-4^T^ (98.4%) and *Paenibacillus xanthinolyticus* 11N27^T^ (98.0%). The pairwise average nucleotide identity and digital DNA-DNA hybridisation values of the JJ-246^T^ genome assembly against publicly available *Paenibacillus* type strain genomes were below 82% and 33%, respectively. The draft genome of JJ-246^T^ shared many putative plant-beneficial functions contributing (PBFC) genes, related to plant root colonisation, oxidative stress protection, degradation of aromatic compounds, plant growth-promoting traits, disease resistance, drug and heavy metal resistance, and nutrient acquisition. The quinone system of strain JJ-246^T^, the polar lipid profile and the major fatty acids were congruent with those reported for members of the genus *Paenibacillus*. JJ-246^T^ was shown to represent a novel species of the genus *Paenibacillus*, for which the name *Paenibacillus plantiphilus* sp. nov. is proposed, with JJ-246^T^ (= LMG 32093^T^ = CCM 9089^T^ = CIP 111893^T^) as the type strain.

**Supplementary Information:**

The online version contains supplementary material available at 10.1007/s10482-023-01852-x.

## Introduction

The genus *Paenibacillus* is a still growing bacterial genus now accommodating more than 280 species (https://lpsn.dsmz.de/genus/paenibacillus) isolated from various sources. Initially proposed by Ash et al. (1993) on the basis of few species it is very clear, that species of *Paenibacillus* can be found in many different habitats, among them plants and their rhizosphere (Elo et al. 2001; Ma et al. 2007; Kim et al. 2009a,b, 2015a,b; Hong et al. 2009; Beneduzi et al. 2010; Zhang et al. 2013, 2015; Wang et al. 2014; Son et al. 2014; Han et al. 2015; Kämpfer et al. 2016, 2017a,b, 2021, 2022; Xin et al. 2017), seeds (Liu et al. 2015) or the phyllosphere (Rivas et al. 2005, 2006). They can be also found in plants as endophytes (Carro et al. 2013; Lai et al. 2015; Kittiwongwattana & Thawai 2015; Gao et al. 2015).

In this context, they may play an important role in their capacity to promote plant growth and form endospores (Grady et al. 2016). Several plant growth promoting traits have been confirmed for several members of the genus *Paenibacillus* (e.g. Wang et al. 2023)*.* Plant growth promoting rhizobacteria (PGPR) are of increasing importance to enhance the efficiency and yield in sustainable agricultural crob production (Grady et al. 2016).

For this purpose we aimed to isolate further new *Paenibacillus* species colonising the rhizosphere and roots of agricultural crop plants which may support efficiently plant growth and agricultural yield production (de Andrade et al. 2023). In this context we characterised the new *Paenibacillus* sp. JJ-246^T^ and studied its genome for the presence of different plant-beneficial function contributing (PBFC) genes, related to plant root colonisation, oxidative stress protection, degradation of aromatic compounds, plant growth-promoting traits, disease resistance, drug and heavy metal resistance, nutrient acquisition, nitrogen fixation, and phosphate solubilisation in addition for its capacity to produce indole-3-acetic acid (IAA).

## Materials and methods

### Isolation and culture conditions

A collection of 1,884 bacterial strains were obtained from field-grown maize plants (*Zea mays*) in Dunbar, Nebraska. The collection was assembled with the purpose of finding strains with beneficial plant properties. Fields in the US Midwest have a history of successfully supporting healthy crops and harvests, and it was surmised that the microbes in these fields may play a role in the overall health of these crops. Once in pure culture, strains could then be tested individually by seed-treatment in a greenhouse assay on agriculturally important crops. Toward this goal, two-week-old maize seedlings were manually uprooted. Roots, approximately 15 cm in length, were separated from the surrounding soil by vigorous shaking such that only the most tightly adhering soil remained. Bacteria were harvested from the root surface by immersing the root in sterile water followed by plating dilutions on Nutrient Agar (Sigma-Aldrich). Strain JJ-246^T^ was initially isolated at Auburn University in this manner. Subsequent cultivation for detailed characterisation of JJ-246^T^ was performed on tryptone soya agar (TSA, Oxoid) at 28 °C for 24 h. The medium was chosen because reference *Paenibacillus* strains used for comparative purpose were routinely cultured under the same conditions.

### 16S rRNA gene sequencing and analysis

For a first phylogenetic placement, the 16S rRNA gene of strain JJ-246^T^ was PCR amplified with primer system Eub9f (5′-GAGTTTGATCMTGGCTCAG-3′) and Eub1492R (5′-ACGGYTACCTTGTTACGACTT-3′) (Lane 1991), and sequenced with the Sanger dideoxy sequencing technology using primers Eub9f and E786F (5′-GATTAGATACCCTGGTAG-3′), respectively, as described previously (Schauss et al. 2015). The sequence was manually processed and corrected based on the electropherograms using MEGA11 (Tamura et al. 2021).

A first phylogenetic assignment was performed by BLAST analysis against the EzBioCloud 16S rRNA gene sequence database including all type strain 16S rRNA gene sequences and blastn blast + v2.13.0 of the NCBI (https://blast.ncbi.nlm.nih.gov) against the curated 16S ribosomal RNA type strain database (update 2023/01/12). The 16S rRNA gene sequences of strain JJ-246^T^ and non-included related type strains were subsequently added to LTP_2020 database of the “All-Species Living Tree'' Project (LTP; Yarza et al. 2008) using ARB release 5.2 (Ludwig et al. 2004). The updated database version LTP_04_2021 (released in September 2021) was used. The imported 16S rRNA gene sequences were aligned in the alignment explorer using the pt server generated for the respective database. The alignment was checked manually before subsequent analyses. The sequence was added to the database tree tree_Ltp_all_04_21 containing all type strain 16S rRNA gene sequences of species described until May 2021 using the parsimony quick ad marked tool of ARB and the gap95_q0_to_q5 filter.

All strains of the resulting cluster and the 16S rRNA gene sequence of the type strain of the type species *P. polymyxa* and next related type strains which clustered with that were included in the analysis. According to previous phylogenetic analysis of the genus *Paenibacillus*, two *Cohnella* type strains were used as outgroup (Kämpfer et al. 2022). The genus is assigned as a neighbouring monophyletic genus. Different phylogenetic trees were calculated which were all based on gene termini 95 to 1475 (*Escherichia coli* numbering, Brosius et al. 1978). This sequence range was covered by all compared sequences. A maximum-likelihood tree was calculated with RAxML v7.04 (Stamatakis 2006), GTR-GAMMA and rapid bootstrap analysis and a maximum parsimony tree with DNAPARS v 3.6 (Felsenstein 2005). Both trees were calculated with 100 re-samplings (bootstrap analysis; Felsenstein 1985) and based on 16S rRNA gene sequences between gene termini 95 to 1446 (*Escherichia coli* numbering, Brosius et al. 1978).

### Genome sequencing and analyses

The whole genome sequencing of strain JJ-246^T^ was carried out with a NextSeq500 instrument using the Nextera XT DNA library preparation kit (Illumina) and a 2 × 150 bp paired-end protocol, yielding 3,948,028 read pairs (192 × average sequencing depth, 361 bp average insert size). Read processing, genome assembly and gene annotation were performed using fq2dna v21.06 (https://gitlab.pasteur.fr/GIPhy/fq2dna).

Pairwise average nucleotide and amino acid identity (ANI and AAI, respectively) values were computed using OGRI_B v1.1 (https://gitlab.pasteur.fr/GIPhy/OGRI) between the draft genome of JJ-246^T^ and every *Paenibacillus* type strain genome selected for the 16S rRNA phylogenetic analysis (when publicly available).

A phylogenetic classification of these genomes was inferred using JolyTree v2.1 (Criscuolo 2019, 2020). An alternative phylogenetic tree was inferred using the 120 conserved phylogenetic markers suggested by Parks et al. (2018). For each locus, gathered amino acid sequences were aligned using MAFFT v7.467 (Katoh and Standley 2013, 2016), and next processed using BMGE v2.0 (Criscuolo and Gribaldo 2010) to select aligned characters that are suited for phylogenetic inference (default options; see Text S1). Maximum likelihood phylogenetic inference from the concatenation of the resulting 120 multiple sequence alignments was carried out using IQ-TREE v2.2.2.2 (Minh et al. 2020) with evolutionary model LG + I + F + R5, derived by minimising the Bayesian information criterion as estimatedby default in IQ-TREE (Kalyaanamoorthy et al. 2017). All phylogenetic trees were drawn using FigTree v1.4.4 (http://tree.bio.ed.ac.uk/software/figtree).

Plant-beneficial function contributing (PBFC) genes (as listed in Cherif-Silini et al. 2019) were searched using tblastn against the draft genome of JJ-246^T^. Biosynthetic gene clusters for secondary metabolites were inferred using antiSMASH v7.0.0beta1-67b538a9 (Blin et al. 2021).

### Physiology and chemotaxonomy

A detailed phenotypic characterisation of strain JJ-246^T^ was performed on the basis of the methods published previously (Kämpfer et al. 2016, 2017a,b, 2021). Cell morphology and motility was observed under a Zeiss light microscope at a magnification of 1000, using cells that had been grown for 3 days at 28 °C on TSA (Oxoid). Gram-staining was performed by the modified Hucker method according to Gerhardt et al. (1994). Oxidase activity was tested with a oxidase reagent text kit following the instructions of the manufacturer (bioMérieux, France). Growth was tested on different agar media including R2A (Oxoid), Nutrient broth agar (NB, Oxoid), TSA, Malt agar (Merck), PYE [0.3% (w/v) yeast extract, and 0.3% (w/v) casein peptone, respectively, 15 g agar L^−1^, pH 7.2], CASO agar (Carl Roth), K7 [0.1% (w/v) of yeast extract, peptone, and glucose, 15 g L^−1^ agar, pH 6.8], medium 65 (M65, according to DSMZ), DEV agar (DEV, Merck), Nutrient agar (NA, Becton Dickinson), Luria Bertani agar (LB, Sigma-Aldrich), Marine agar 2216 (MA, Becton Dickinson), Columbia agar with sheep blood (Oxoid), and MacConkey agar (Oxoid). Growth was evaluated after 48 h incubation at 28 °C. Temperature dependent growth was determined on Columbia agar with sheep blood at 4, 10, 15, 20, 25, 28, 30, 36, 45, 50, and 55 °C. pH and salinity dependent growth was tested in R2A broth incubated at 28 °C. The pH was adjusted to pH 4.5 to 10.5 (1 pH unit intervals incrementing) using HCl and NaOH. For salinity dependent growth 1 to 8% (w/v) NaCl was added (in 1% intervals incrementing). Growth was monitored after 72 h of incubation.

Further physiological characterisation of the strains was performed with the API 20NE and API ZYM test systems according to the instruction of the manufacturer (bioMérieux) and with methods described by Kämpfer et al. (1991, 1993) and Kämpfer (1990). All tests were incubated at 28 °C.

For analyses of quinones and polar lipids, cells were grown in trypton soya broth at 30 °C for 48 h. Quinones were extracted as described by Minnikin et al. (1984) and by Wiertz et al. (2013) and analysed with an Agilent 1260 infinity HPLC system. Polar lipids were extracted and analysed by thin-layer chromatography (TLC) according to Minnikin et al. (1984). TLC plates were stained with ninhydrin for detection of aminolipids and with molybdenum blue reagent for detection of phospholipids. All polar lipids were visualised with sulfuric acid and heating the plates on 140 °C.

The fatty acids were extracted and analysed as described by Kämpfer and Kroppenstedt (1996). Strains were grown under identical conditions (TSA after 72 h incubation at 28 °C) and the cells for extractions were taken from colonies of the same size. Fatty acids were identified with the Sherlock version 2.11, TSBA40 Rev. 4.1.

## Results and discussion

### Molecular and genome characteristics

The final corrected 16S rRNA gene sequence of strain JJ-246^T^ (OQ300222) had a size of 1,479 nt and spanned gene termini 8 to 1,475 (numbering according to the *Escherichia coli rrnB* sequence published by Brosius et al. 1978). Based on the 16S rRNA gene sequences, strain JJ-246^T^ was placed within the cluster of *Paenibacillus* type strains determined as the next related species by BLAST analyses. Strain JJ-246^T^ showed highest 16S rRNA gene sequence similarity to the type strain of *Paenibacillus oenotherae* (98.4%), followed by the type strain of *Paenibacillus xanthinilyticus* (98.0%); sequence similarities to all other type strains were below 97%.

Several phylogenetic trees were calculated and several outgroup strains were tested. The genus *Cohnella* is monophyletic and well distinguishable to members of the paraphyletic genus *Paenibacillus*, for that reason it gave a well supported outgroup taxon. Independent of the applied treeing method, strain JJ-246^T^ showed a tendency to cluster with the type strains of *P. oenotherae* and *P. xanthinilyticus* (70% bootstrap support; Fig. S1).

The draft genome of strain JJ-246^T^ was made of 6,062,127 bps on 102 contigs (N50, 147,865) with G + C content of 49.25 mol%. A total of 5,293 coding sequences and 64 tRNA was inferred. Genome sequence authenticity was assessed by aligning the 16S rRNA segment derived from Sanger sequencing (OQ300222) against the *de novo* assembly (CAKMMF000000000) using blastn, leading to 99.73% pairwise sequence similarity.

**Fig. 1:**
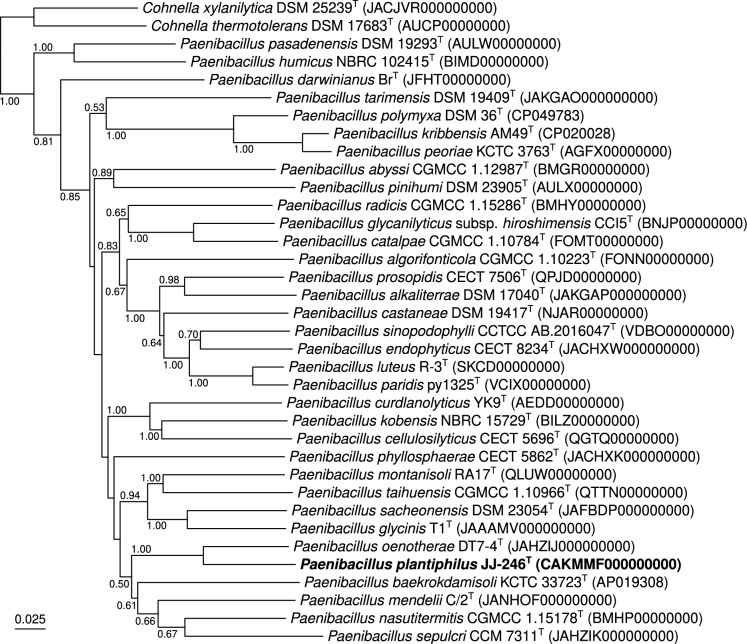
Whole-genome-based tree showing the phylogenetic placement of strain JJ-246^T^ among type strains of closely related *Paenibacillus* species. This minimum evolution tree was inferred using JolyTree (https://gitlab.pasteur.fr/GIPhy/JolyTree). Two publicly available *Cohnella* type strain genomes were used as outgroup. The genome sequence accession is specified between parentheses next after each taxon name. Branch supports were assessed by the rate of elementary quartets, as estimated by JolyTree (only supports > 0.5 were specified). Bar, 0.025 nucleotide substitutions per site

Average nucleotide and amino acid identity (ANI and AAI, respectively) values are reported in Table S1, together with the associated digital DNA-DNA hybridisation (dDDH) values (formula 2; https://ggdc.dsmz.de). All these estimated pairwise similarity values are far below the commonly admitted species delineation cutoffs (ANI, 95%; AAI, 96%; dDDH, 70%). Phylogenomic classifications confirmed that strain JJ-246^T^ formed a stable cluster (100% branch support) with the type strain of *P. oenotherae* (Fig. 1; Fig. S2).

The draft genome of JJ-246^T^ shares different plant-beneficial function contributing (PBFC) genes, related to plant root colonisation (Table S2), oxidative stress protection (Table S3), degradation of aromatic compounds (Table S4), plant growth-promoting traits (Table S5), disease resistance (Table S6), drug and heavy metal resistance (Table S7), and nutrient acquisition (Table S8). Contrary to many *Paenibacillus* strains (e.g. Xie et al. 2014), the genome of JJ-246^T^ does not seem to share particular genes related to nitrogen fixation. However, it contains genes related to phosphate solubilisation (Table S8), most of them being grouped into a unique cluster (CAH1206316-CAH1206402) comparable to a phosphate-specific transport (*tsp*) operon (e.g. Li et al. 2020). Phophorus (P) is an essential macronutrient for plants which is limited in bioaviable forms in soil. Solubilisation of P by PGPR is an sufficient process to enhance the P availability for the uptake by plant roots and improve thereby crob yield (Din et al*.* 2020). The genome of JJ-246^T^ also shares a gene cluster *trpEDCFBA* (CAH1218260-CAH1218273; Table S5), related to the production of indole-3-acetic acid (IAA), an important phytohormone known to stimulate plant growth (e.g. Yuan et al. 2022; Shao et al. 2015).

Finally, a total of 16 biosynthetic gene clusters involved in secondary metabolite production were found, including eight non-ribosomal peptide synthetase, four polyketide synthase, three agrD-like cyclic lactone autoinducer peptides, two IucA/Iuc-like siderophores, one lasso peptide, one heterocyst glycolipid synthase-like polyketide synthase, one terpene and one resorcinol clusters (Table S9). Interestingly, two of them are quite similar to paenibacterin and paeninodin biosynthetic gene clusters (60% and 80% gene similarity, respectively) already described in other *Paenibacillus* strains and involved in the production of broad-spectrum antimicrobial compounds (Huang et al. 2014; Zhu et al. 2016).

### Phenotype

The results of the physiological characterisation are given in Table 1 and in the species description. Strain JJ-246^T^ can be differentiated from the type strains of the most closely related species with a battery of selected physiological tests. Strain JJ-246^T^ was not able to hydrolyse starch and to utilise L-arabinose, sucrose, amygdalin in contrast to *P. oenotherae* DLE-12^T^ and *P. xanthinilyticus* 11N27^T^, which both showed positive reactions in these tests. In addition, strain JJ-246^T^ did not produce α-galactosidase which differentiated the strain from *P. oenotherae* DLE-12^T^ and was able to grow at 37 °C which differentiated the strain from *P. xanthinilyticus* 11N27^T^. More differentiating tests are shown in Table 1. The polar lipid profile consisted of diphosphatidylglycerol (DPG), phosphatidylethanolamine (PE), phosphatidylglycerol (PG), phosphatidylserine (PS) and one additional unidentified aminophospholipid (APL) (Fig. S3). The type strain of *P. oenotherae* DLE-12^T^ also revealed diphosphatidylglycerol (DPG), phosphatidylethanolamine (PE), phosphatidylglycerol (PG) as major lipids, but did not produce phosphatidylserine (PS), but instead three unidentified aminophospholipids, an unidentified aminolipid, an unidentified phospholipid and two unidentified lipids (Kim et al. 2015b). The polar lipids present in strain *P. xanthinilyticus* 11N27^T^ were also phosphatidylethanolamine (PE), two unknown phospholipids, two unknown aminolipids, one unknown aminophospholipid, and two unknown polar lipids that were not stainable with any of the specific spray reagents applied to detect a phosphate, an amino-, or a sugar moiety (Kim et al. 2015a). It is quite likely that among the two unidentified phospholipids also diphosphatidylglycerol (DPG) and phosphatidylglycerol (PG) are produced, which are typical for *Panibacillus* species.

**Table 1:** Differential characteristics of strain JJ-246^ T^ and phylogenetically related species of the genus *Paenibacillus*

Characteristic	1	2	3
Oxidase	+	+	−
Growth at/in			
37 °C	+	+	−
1% NaCl	+	+	−
Hydrolysis of:			−
Starch	−	+	+
API 20E reactions			
L-arabinose	−	+	+
D-mannitol	−	−	+
N-acetyl-D-glucosamine	−	(+)	-
Sucrose	−	+	+
Amygdalin	−	+	+
Melibiose	−	+	−
Enzyme activities (API ZYM)			
Acid phosphatase	−	−	+
*α*-Galactosidase	−	+	−
*α*-Glucosidase	−	−	+

Strains: 1, JJ-246^ T^; 2, *Paenibacillus oenotherae* DLE-12^T^; 3, *Paenibacillus xanthinilyticus* 11N27^T^. Data for taxa 2 and 3 from Kim et al. (2015a) and Kim et al. (2015b), respectively, obtained with standardised tests. + , Positive; −, negative; (+), weakly positive

The dominating quinone of strain JJ-246^T^ was menaquinone MK-7 (97%)  Minor amounts of menaquinone MK-8 (3%) were also detected. Presence of menaquinone MK-7 as well as the polar lipid profile are in agreement with the characteristics of other species of the genus *Paenibacillus*, also for *P. xanthinilyticus* and *P. oenotherae* (Kim et al. 2015a,b).

The fatty acids comprised mainly iso- and anteiso-branched fatty acids, and the fatty acid profile was very similar to that of the most closely related *Paenibacillus* species, which is also quite typical for *Paenibacillus* species, which are quite similar with respect to fatty acid profiles. Strain JJ-246^T^ was able to produce C_15:0_ which was not detected in *P. oenotherae* DLE-12^T^ and *P. xanthinilyticus* 11N27^T^*.* For the majority of other fatty acids in most cases only minor quantitative differences could be found. The detailed fatty acid profile obtained from cells grown is shown in Table S10.

## Conclusion

On the basis of genotypic and phenotypic features a novel species of the genus *Paenibacillus* is proposed for which the name *Paenibacillus plantiphilus* is proposed with JJ-246^T^ as type strain. The strain was isolated from the rhizosphere of * Zea mays* indicating its potential to grow efficient in the rhizosphere of agricultural plants. Genome analyses showed the potential of the strain to represent a novel PGPR.

### Description of *Paenibacillus plantiphilus* sp. nov.

*Paenibacillus plantiphilus* (plan.ti'phi.lus. L. fem. n. *planta*, a plant; Gr. masc. adj. *philos*, loving; N.L. masc. adj. *plantiphilus*, plant-loving).

Cells (with rounded ends) stain Gram-positive. No chains or filaments could be observed after growth on TSA at 28 °C for 48 h. Cells were 2.0-3.0 µm in length and 0.8-1.0 µm in width) and showed no motility. Endospores were not observed. No other cell inclusions could be detected. Colonies grown on TSA (Oxoid) at 28°C after 48 h of incubation are circular, convex and beige with a shiny appearance and an average diameter of 2 to 3 mm. Growth at 28 °C on R2A (Oxoid), CASO agar (Carl Roth), K7, M65, DEV agar (DEV, Merck), NA (Becton Dickinson), LB (Sigma-Aldrich), and Columbia agar with sheep blood (Oxoid). No growth on MA (Becton Dickinson), NB (Oxoid), Malt agar (Merck), and MacConkey agar (Oxoid).

Good growth on CASO agar between 10 and 45 °C (weak) (optimal growth at 25–30 °C), but not at 4 °C and 50 °C. Optimal pH for growth in R2A broth at 28 °C is pH 7–8; growth occurs between pH 5.5–10.5. Optimal growth in R2A broth at 28 °C without and in the presence of 1% NaCl, growth occurs between 0 to 1% NaCl, but not at 2% or above. Tests for catalase and oxidase activities are positive. According to API 20 NE, positive for the assimilation of D-glucose, L-arabinose and D-maltose, but negative in the activities of *β*-glucosidase (esculin hydrolysis), PNPG beta-galactosidase, indole production, D-glucose fermentation, arginine dihydrolase, urease, gelatin hydrolysis and the assimilation of D-mannitol, N-acetylglucosamine, D-maltose, capric acid, adipic acid, malic acid, trisodium citrate, and phenylacetic acid. According to API 20E and to tests of Kämpfer et al. (1991), negative in acid formation of D-glucose, L-arabinose, D-mannose, D-mannitol, N-acetylglucosamine, D-maltose, sucrose, amygdalin, inositol, melibiose.

According to API ZYM, positive for naphtol-AS-BI-phosphohydrolase and negative for leucine arylamidase, acid phosphatase, *β*-galactosidase, alkaline phosphatase, esterase (C4), esterase lipase (C8), lipase (C14), valine arylamidase, cystine arylamidase, trypsin, *α*-chymotrypsin, *α-*galactosidase, *β*-glucuronidase, *α*-glucosidase, *β*-glucosidase, N-acetyl-*β*-glucosaminidase, *α*-mannosidase, and *α*-fucosidase.

Some sugars or sugar-related compounds were utilised by strain JJ-246^T^ according to the method of Kämpfer et al. (1991): L-arabinose (weak), arbutin, D-cellobiose, D-galactose, glucose (weak), D-maltose, D-melibiose, sucrose, D-trehalose, D-xylose and D-maltitol are utilised as sole sources of carbon. D-gluconate, ribose, D-fructose, i-inositol, D-mannose, salicin, D-sorbitol, acetate, N-acetyl-D-glucosamine, cis-aconitate, trans-aconitate, adipate, D-adonitol, 4-aminobutyrate, azelate, citrate, itaconate, malate, mesaconate, 2-oxoglutarate, propionate, putrescine, pyruvate and L-rhamnose are not utilised as sole carbon source.

The quinone system is dominated by menaquinone MK-7. The polar lipid profile is composed of the major lipids diphosphatidylglycerol, phosphatidylglycerol, phosphatidylethanolamine, phosphatidylserine and one additional unidentified aminophospholipid (APL).

Major fatty acids are anteiso C_15:0_ and iso C_15:0_. The genomic DNA G + C content is 49.25 mol% (based on the genome sequence).

The type stain JJ-246^T^ (= LMG 32093^T^ = CCM 9089^T^ = CIP 111893^T^) was isolated from the root surface of a field-grown corn plant in Dunbar, Nebraska USA. The genome sequence of the type strain is available under accession number CAKMMF00000000, and the 16S rRNA gene sequence under accession number OQ300222.

## Supplementary Information

Below is the link to the electronic supplementary material.

Supplementary file1 (PDF 1532 KB)

## Data Availability

The 16S rRNA gene and the complete genome sequences of strain JJ-246^T^ have been deposited under the GenBank/EMBL/DDBJ accession numbers OQ300222 and CAKMMF000000000, respectively.
