# Supplementary material for: Paenibacillus plantiphilus sp. nov. from the plant environment of Zea mays
Source: Antonie Van Leeuwenhoek. 2023 Jun 20;116(9):883–92. doi: 10.1007/s10482-023-01852-x (PMC10371898; doi:10.1007/s10482-023-01852-x)
Supplement: Supplementary file 1 — Supplementary file1 (PDF 1532 KB) [file 10482_2023_1852_MOESM1_ESM.pdf]

Antonie van Leeuwenhoek

Paenibacillus plantiphilus sp. nov. from the plant environment of *Zea mays*

Peter Kämpfer [1,*], Andre Lipski [2], Lucie Lamothe [3,4], Dominique Clermont [5], Alexis Criscuolo [4], John A. McInroy [6], Stefanie P. Glaeser [1]

[1] Institut für Angewandte Mikrobiologie, Universität Giessen, Germany

[2] Institut für Ernährungs- und Lebensmittelwissenschaften, Lebensmittelmikrobiologie und –hygiene, Rheinische Friedrich-Wilhelms-Universität Bonn, Germany

[3] CNRS, Institut Français de Bioinformatique, IFB-core, UMS 3601, Evry, France

[4] Institut Pasteur, Université de Paris, Bioinformatics and Biostatistics Hub, F-75015 Paris, France

[5] Institut Pasteur, Université de Paris, CIP - Collection of Institut Pasteur, F-75015 Paris, France

[6] Department of Entomology and Plant Pathology, Auburn University, Alabama, USA

[*] Corresponding author (peter.kaempfer@umwelt.uni-giessen.de)

Supplementary Information

**Table S1**	Overall genome relatedness indices
**Tables S2-S8**	Putative plant-beneficial function contributing genes
**Table S9**	Sixteen gene clusters for secondary metabolites predicted by antiSMASH
**Table S10**	Cellular fatty acid profiles
**Figure S1**	16S rRNA phylogenetic tree
**Figure S2**	Multiple-gene phylogenetic tree
**Figure S3**	Polar lipid profile
**Text S1**	Bioinformatic command line summary

Table S1. Overall genome relatedness indices between strain JJ-246^T and next related *Paenibacillus* type strains

type strain	genome accession	% ANI [CI]	% AAI [CI]	% dDDH [CI]
P. oenotherae DT7-4^T	JAHZIJ000000000	81.18 [80.94-81.43]	84.23 [83.87-84.59]	25.60 [23.3-28.1]
P. montanisoli RA17^T	QLUW00000000	72.50 [72.18-72.74]	72.34 [71.87-72.87]	19.00 [16.9-21.4]
P. sepulcri CCM 7311^T	JAHZIK000000000	72.46 [72.14-72.76]	72.83 [72.25-73.47]	20.40 [18.2-22.8]
P. nasutitermitis CGMCC 1.15178^T	BMHP00000000	72.41 [72.14-72.66]	72.34 [71.79-72.91]	19.80 [17.6-22.2]
P. mendelii C/2^T	JANHOF000000000	72.37 [72.09-72.64]	72.44 [71.94-73.01]	18.90 [16.7-21.3]
P. baekrokdamisoli KCTC 33723^T	AP019308	72.24 [71.94-72.49]	73.09 [72.56-73.57]	19.20 [17.0-21.6]
P. taihuensis CGMCC 1.10966^T	QTTN00000000	72.23 [71.91-72.54]	71.82 [71.11-72.36]	18.70 [16.5-21.1]
P. glycinis T1T^T	JAAAMV000000000	72.10 [71.79-72.39]	71.57 [71.04-72.07]	18.70 [16.5-21.0]
P. sacheonensis DSM 23054^T	JAFBDP000000000	72.05 [71.77-72.36]	71.36 [70.84-71.96]	19.00 [16.8-21.3]
P. phyllosphaerae CECT 5862^T	JACHXK000000000	71.45 [71.19-71.74]	70.38 [69.84-70.91]	18.80 [16.6-21.1]
P. darwinianus BrT^T	JFHT00000000	71.19 [70.86-71.50]	70.42 [69.77-71.06]	18.20 [16.0-20.5]
P. abyssi CGMCC 1.12987^T	BMGR00000000	70.82 [70.54-71.13]	69.86 [69.22-70.42]	18.90 [16.7-21.3]
P. alkaliterrae DSM 17040^T	JAKGAP000000000	70.46 [70.10-70.73]	68.79 [68.36-69.40]	19.40 [17.2-21.8]
P. prosopidis CECT 7506^T	QPJD00000000	70.40 [70.12-70.70]	68.84 [68.25-69.34]	19.40 [17.2-21.8]
P. algorifonticola CGMCC 1.10223^T	FONN00000000	70.35 [70.06-70.63]	67.06 [66.59-67.55]	20.00 [17.8-22.4]
P. radicis CGMCC 1.15286^T	BMHY00000000	70.26 [69.97-70.60]	67.93 [67.40-68.50]	19.10 [16.9-21.5]
P. curdlanolyticus YK9^T	AEDD00000000	70.16 [69.81-70.46]	67.56 [67.00-68.01]	21.50 [19.3-24.0]
P. kobensis NBRC 15729^T	BILZ00000000	70.15 [69.92-70.42]	67.14 [66.49-67.66]	19.00 [16.8-21.4]
P. catalpae CGMCC 1.10784^T	FOMT00000000	70.12 [69.87-70.44]	67.98 [67.43-68.47]	19.40 [17.2-21.8]
P. glycanilyticus subsp. *hiroshimensis* CCI5^T	BNJP00000000	70.10 [69.85-70.39]	67.71 [67.17-68.25]	18.60 [16.4-20.9]
P. luteus R-3^T	SKCD00000000	70.10 [69.78-70.33]	67.89 [67.34-68.43]	19.20 [17.0-21.6]
P. castaneae DSM 19417^T	FNDY00000000	69.99 [69.70-70.30]	68.34 [67.83-68.82]	20.30 [18.1-22.8]
P. endophyticus CECT.8234^T	CAJVAS000000000	69.98 [69.70-70.27]	67.99 [67.46-68.49]	19.40 [17.2-21.8]
P. paridis py1325^T	VCIX00000000	69.88 [69.59-70.14]	67.62 [67.10-68.15]	18.40 [16.3-20.8]

Table S1. (cont.)

type strain	genome accession	% ANI [CI]	% AAI [CI]	% dDDH [CI]
P. sinopodophylli CCTCC AB.2016047^T	VDBO00000000	69.84 [69.55-70.12]	67.59 [67.13-68.07]	19.40 [17.2-21.8]
P. cellulosilyticus CECT 5696^T	QGTQ00000000	69.84 [69.53-70.12]	66.92 [66.38-67.50]	19.70 [17.5-22.2]
P. pinihumi DSM 23905^T	AULX00000000	69.79 [69.50-70.07]	67.21 [66.69-67.80]	19.20 [17.0-21.6]
P. tarimensis DSM 19409^T	JAKGAO000000000	69.71 [69.36-69.98]	67.46 [66.84-68.01]	19.10 [16.9-21.5]
P. humicus NBRC 102415^T	BIMD00000000	69.60 [69.30-69.88]	66.0 [65.45-66.62]	20.30 [18.1-22.7]
P. pasadenensis DSM 19293^T	AULW00000000	69.20 [68.95-69.47]	65.75 [65.18-66.32]	18.90 [16.7-21.2]
P. peoriae KCTC 3763^T	AGFX00000000	68.22 [67.94-68.54]	63.62 [62.92-64.20]	23.20 [20.9-25.7]
P. kribbensis AM49^T	CP020028	68.13 [67.83-68.40]	63.77 [63.03-64.38]	29.20 [26.8-31.7]
P. polymyxa ATCC 842^T	CP024795	67.75 [67.43-68.09]	63.46 [62.91-64.01]	30.40 [28.0-32.9]

Table S2. Genes of strain JJ-246^T potentially related with root colonisation/growth promoting factors

accession	gene	annotated product	putative function
CAH1192742	minJ	Cell division topological determinant	Swarming motility
CAH1192746	ctpB	Carboxy-terminal processing protease	
CAH1192753	ftsX	Cell division protein	
CAH1192948	hag_1	Flagellin	
CAH1193004	hag_2		
CAH1193011	-	-	
CAH1193022	-	-	
CAH1193173	flgG_1	Flagellar basal-body rod protein	
CAH1193178	flgG_2		
CAH1197818	flhF	Flagellar biosynthesis protein	
CAH1197820	flhA	Flagellar biosynthesis protein	
CAH1197822	flhB_1	Flagellar biosynthetic protein	
CAH1197842	flgG_3		
CAH1197846	-	-	
CAH1197856	flgC	Flagellar basal-body rod protein	
CAH1197857	flgB	Flagellar basal body rod protein	
CAH1199325	swrC_1	Swarming motility protein	
CAH1202728	swrC_2		
CAH1204126	swrC_3		
CAH1207797	swrC_4		
CAH1210116	swrC_5		
CAH1192731	pomA_1	Chemotaxis protein	Chemotaxis ability
CAH1192734	lafU	Chemotaxis protein	
CAH1192962	fliS	Flagellar secretion chaperone	
CAH1192969	fliD	Flagellar hook-associated protein 2	
CAH1193722	cheR_1	Chemotaxis protein methyltransferase	
CAH1197688	cheB_1	Protein-glutamate methylesterase/protein-glutamine glutaminase	
CAH1197806	cheD	Chemoreceptor glutamine deamidase	
CAH1197808	cheC	CheY-P phosphatase	
CAH1197810	cheW_1	Chemotaxis protein	
CAH1197812	cheA_1	Chemotaxis protein	
CAH1197814	cheB3	Protein-glutamate methylesterase/protein-glutamine glutaminase of group 3 operon	
CAH1197824	fliR	Flagellar biosynthetic protein	
CAH1197826	-	-	
CAH1197828	fliP	Flagellar biosynthetic protein	
CAH1197832	cheY	Chemotaxis protein	
CAH1197834	-	-	
CAH1197836	fliM	Flagellar motor switch protein	
CAH1197838	-	-	
CAH1197848	-	-	
CAH1197850	-	-	
CAH1197851	yscN	putative ATP synthase	
CAH1197852	-	-	
CAH1197853	fliG	Flagellar motor switch protein	
CAH1197854	-	-	
CAH1197855	fliE	Flagellar hook-basal body complex protein	
CAH1201351	-	-	
CAH1201354	-	-	
CAH1201755	cheB_4		
CAH1206778	cheW_2		
CAH1206781	-	-	
CAH1206784	cheB_7		
CAH1214917	cheR_2		
CAH1214927	cheW_3		
CAH1214932	cheA_2		
CAH1218242	cheR_3		
CAH1221658	-	-	
CAH1222116	pomA_2		
CAH1222123	motB	Motility protein B	
CAH1190331	xerH	Tyrosine recombinase	Rhizosphere colonization
CAH1190380	xerC_1	Tyrosine recombinase	
CAH1208743	xerS	Tyrosine recombinase	
CAH1205352	xerC_2		
CAH1210394	xerD	Tyrosine recombinase	
CAH1226055	xerC_4		

Table S2. (cont.)

accession	gene	annotated product	putative function
CAH1192899	epsM	putative acetyltransferase	Exopolysaccharide biosynthesis
CAH1206217	pseB	UDP-N-acetylglucosamine 4,6-dehydratase(inverting)	
CAH1206823	ywqC_1	putative capsular polysaccharide biosynthesis protein	
CAH1206826	ywqD_1	Tyrosine-protein kinase	
CAH1206829	pglF	UDP-N-acetyl-alpha-D-glucosamine C6 dehydratase	
CAH1206838	epsF_2	Putative glycosyltransferase	
CAH1206853	mshA_5	D-inositol-3-phosphate glycosyltransferase	
CAH1206859	dapH_2	2,3,4,5-tetrahydropyridine-2,6-dicarboxylate N-acetyltransferase	
CAH1206864	epsN	Putative pyridoxal phosphate-dependent aminotransferase	
CAH1211029	ywqD_2		
CAH1211032	ywqC_2		

Table S3. Genes of strain JJ-246^T potentially related with plant protection from oxidative stress

accession	gene	annotated product	putative function
CAH1190071	ahpC	Alkyl hydroperoxide reductase C	Antioxidant enzymes
CAH1193635	ohrR	Organic hydroperoxide resistance transcriptional regulator	
CAH1193631	gpx1	Hydroperoxy fatty acid reductase	
CAH1193635	ohrR	Organic hydroperoxide resistance transcriptional regulator	
CAH1195994	oxyR	Hydrogen peroxide-inducible genes activator	
CAH1209045	hmp	Flavohemoprotein	
CAH1217501	katA_1	Catalase	
CAH1218059	bsaA	Glutathione peroxidase	
CAH1218572	sodA	Superoxide dismutase [Mn]	
CAH1218737	-	-	
CAH1218847	bcp	Putative peroxiredoxin	
CAH1219126	-	Manganese catalase	
CAH1224785	ywrD	Glutathione hydrolase-like YwrD proenzyme	
CAH1224825	katA_2		
CAH1225917	tpx	Thiol peroxidase	

Table S4. Genes of strain JJ-246^T potentially related with degradation of aromatic compounds

accession	gene	annotated product	putative function
CAH1190231	yodC	Putative NAD(P)H nitroreductase	2-Methylhydroquinone and catechol resistance
CAH1201805	azoR4	FMN-dependent NADH-azoreductase 4	
CAH1208040	mhqR	HTH-type transcriptional regulator	
CAH1208042	mhqP	Putative oxidoreductase	
CAH1215722	azoR2	FMN-dependent NADH-azoreductase 2	
CAH1224810	mhqD	Putative hydrolase	
CAH1224815	mhqO	Putative ring-cleaving dioxygenase	
CAH1224820	mhqN	Putative NAD(P)H nitroreductase	

Table S5. Genes of strain JJ-246^T potentially related with plant growth-promoting traits

accession	gene	annotated product	putative function
CAH1189953	aroF	Phospho-2-dehydro-3-deoxyheptonate aldolase	Auxin biosynthesis
CAH1190084	ilvB	Acetolactate synthase large subunit	
CAH1190161	-	-	
CAH1193644	bioK	L-Lysine--8-amino-7-oxononanoate transaminase	
CAH1209483	patB	Cystathionine beta-lyase	
CAH1210263	accC	Biotin carboxylase	
CAH1211493	trpS	Tryptophan--tRNA ligase	
CAH1216324	aldH1	4,4'-diaponeurosporen-aldehyde dehydrogenase	
CAH1218251	aroC	Chorismate synthase	
CAH1218260	trpE	Anthranilate synthase component 1	
CAH1218263	trpD	Anthranilate phosphoribosyltransferase	
CAH1218266	trpC	Indole-3-glycerol phosphate synthase	
CAH1218270	trpF	N-(5'-phosphoribosyl)anthranilate isomerase	
CAH1218273	trpB	Tryptophan synthase beta chain	
CAH1218276	trpA	Tryptophan synthase alpha chain	
CAH1221166	ecdB	putative UbiX-like flavin prenyltransferase	
CAH1221192	mtrB	Transcription attenuation protein	
CAH1195116	miaA	tRNA dimethylallyltransferase	Cytokinin biosynthesis
CAH1197732	miaB	tRNA-2-methylthio-N(6)-dimethylallyladenosine synthase	
CAH1189969	nrgA_1	Ammonium transporter	Nitric oxide production
CAH1193603	nasE_1	Assimilatory nitrite reductase [NAD(P)H] small subunit	
CAH1193606	nasD_1	Nitrite reductase [NAD(P)H]	
CAH1204459	nrgA_2		
CAH1216382	nasD_2		
CAH1216392	nasE_2		
CAH1216397	nasD_3		
CAH1221763	nasD_4		
CAH1221771	-	-	
CAH1221775	nirC_1	Nitrite transporter	
CAH1221779	nirC_2		
CAH1193079	metK	S-adenosylmethionine synthase	Polyamines biosynthesis
CAH1199353	speE_1	Polyamine aminopropyltransferase	
CAH1199355	speB	Agmatinase	
CAH1204148	speH	S-adenosylmethionine decarboxylase proenzyme	
CAH1210092	speA_1	Arginine decarboxylase	
CAH1217943	speE_1		
CAH1222741	speA_2		

Table S6. Genes of strain JJ-246^T potentially related with disease resistance

accession	gene	annotated product	putative function
CAH1190082	ilvC	Ketol-acid reductoisomerase (NAD(+))	Acetoine and 2,3-butandiol biosynthesis
CAH1190083	ilvH	Acetolactate synthase small subunit	
CAH1190084	ilvB	Acetolactate synthase large subunit	
CAH1202582	ilvD_1	Dihydroxy-acid dehydratase	
CAH1208876	ilvD_2		
CAH1216961	ilvG	Acetolactate synthase isozyme 2 large subunit	
CAH1217786	ilvA	L-threonine dehydratase biosynthetic	
CAH1195427	gabR_1	HTH-type transcriptional regulatory protein	GABA biosynthesis
CAH1198983	gabR_2		
CAH1211373	gabR_3		
CAH1215790	gabR_4		
CAH1218668	dat	Diaminobutyrate—2-oxoglutarate aminotransferase	

Table S7. Genes of strain JJ-246^T potentially related with drug and heavy metal resistances

accession	gene	annotated product	putative function
CAH1190102<br>CAH1215299	-<br>fosB	-<br>Metallothiol transferase	Fosfomycin resistance
CAH1195106<br>CAH1199351	pbpF<br>pbpG	Penicillin-binding protein 1F<br>Penicillin-binding protein 2D	Penicillin resistance
CAH1210100<br>CAH1223475	tetA_1<br>tetA_2	Tetracycline resistance protein, class C	Tetracycline resistance
CAH1203592<br>CAH1203596<br>CAH1203599	arsC<br>arsB_1<br>aseR	Arsenate reductase<br>Arsenical pump membrane protein<br>HTH-type transcriptional repressor	Arsenic detoxification
CAH1195212<br>CAH1197635<br>CAH1197715<br>CAH1203883<br>CAH1204092<br>CAH1209102<br>CAH1214429<br>CAH1222169	cadA<br>-<br>copA<br>aniA<br>sasA_11<br>ricR<br>kdpB_1<br>kdpB_2	Cadmium-transporting ATPase<br>-<br>Copper-exporting P-type ATPase<br>Copper-containing nitrite reductase<br>Adaptive-response sensory-kinase<br>Copper-sensing transcriptional repressor<br>Potassium-transporting ATPase ATP-binding subunit	Copper resistance
CAH1195075	-	-	Aluminium resistance
CAH1192843<br>CAH1207005<br>CAH1207009<br>CAH1212692<br>CAH1218090<br>CAH1222639	cspB_1<br>crcB_1<br>crcB_2<br>cspB_2<br>cspB_3<br>cspB_4	Cold shock protein<br>Putative fluoride ion transporter	Camphor resistance
CAH1198866<br>CAH1198874<br>CAH1200535<br>CAH1207849<br>CAH1207878<br>CAH1207884<br>CAH1207889<br>CAH1207897<br>CAH1207903	-<br>-<br>-<br>-<br>-<br>-<br>yceD_1<br>yceD_2<br>yceC	-<br>-<br>-<br>-<br>-<br>-<br>General stress protein 16U<br><br>Stress response protein SCP2	Tellurite resistance
CAH1193585	czcD	Cadmium, cobalt and zinc/H(+)-K(+) antiporter	Cation Diffusion Facilitator Transporters
CAH1205056<br>CAH1210169<br>CAH1210177<br>CAH1210378<br>CAH1211575<br>CAH1211579<br>CAH1215201	perR<br>znuB<br>mntR<br>fur<br>mntC<br>mntD<br>zur	Peroxide operon regulator<br>High-affinity zinc uptake system membrane protein<br>HTH-type transcriptional regulator<br>Ferric uptake regulation<br>Manganese transport system membrane protein<br>Manganese transport system membrane protein<br>Zinc-specific metallo-regulatory protein	Metalloregulators
CAH1205213<br>CAH1205219	ndoA<br>ndoAI	Endoribonuclease EndoA<br>Antitoxin EndoAI	Toxin-Antitoxin Module response to stress
CAH1207838<br>CAH1208723	fsr_1<br>fsr_2	Fosmidomycin resistance protein	Fosmidomycin resistance

Table S7. (cont.)

accession	gene	annotated product	putative function
CAH1190154	lnrL_1	Linearmycin resistance ATP-binding protein	Daunorubicin/ Linearmycin resistance
CAH1197723	drrA_1	Daunorubicin/doxorubicin resistance ATP-binding protein	
CAH1198760	drrA_2		
CAH1200588	drrA_3		
CAH1200604	lnrL_3		
CAH1200729	drrA_4		
CAH1201910	lnrL_4		
CAH1210132	lnrL_6		
CAH1212567	lnrL_7		
CAH1214710	lnrL_8		
CAH1226303	lnrL_10		

Table S8. Genes of strain JJ-246^T potentially related with nutrient acquisition

accession	gene	annotated product	putative function
CAH1201814	-	-	Mineral phosphate solubilization
CAH1202670	-	-	
CAH1206316	phoR	Alkaline phosphatase synthesis sensor protein	
CAH1206319	phoP	Alkaline phosphatase synthesis transcriptional regulatory protein	
CAH1206370	pstS1	Phosphate-binding protein	
CAH1206373	-	-	
CAH1206376	-	-	
CAH1206382	pstB3_1	Phosphate import ATP-binding protein	
CAH1206385	phoU_1	Phosphate-specific transport system accessory protein	
CAH1206393	-	-	
CAH1206396	pstA	Phosphate transport system permease protein	
CAH1206399	pstB3_2		
CAH1206402	phoU_2		
CAH1212370	phnE	Phosphate-import permease protein	
CAH1212377	-	-	
CAH1212389	-	-	
CAH1202585	ureA	Urease subunit gamma	Urea utilization
CAH1202588	ureB1	Urease subunit beta 1	
CAH1202591	ureC	Urease subunit alpha	
CAH1202594	ureF	Urease accessory protein	
CAH1202597	ureG	Urease accessory protein	
CAH1202600	ureH	Urease accessory protein	
CAH1189994	frdB	Fumarate reductase iron-sulfur subunit	Exoenzyme and organic acid biosynthesis
CAH1189995	frdA	Fumarate reductase flavoprotein subunit	
CAH1190030	fumC	Fumarate hydratase class II	
CAH1197864	sucD	Succinate--CoA ligase [ADP-forming] subunit alpha	
CAH1197865	sucC	Succinate--CoA ligase [ADP-forming] subunit beta	
CAH1203308	pdhA	Pyruvate dehydrogenase E1 component subunit alpha	
CAH1206993	gndA	6-phosphogluconate dehydrogenase, NADP(+)-dependent, decarboxylating	
CAH1209549	kdgA_1	KHG/KDPG aldolase	
CAH1209675	ppc	Phosphoenolpyruvate carboxylase	
CAH1211044	gltB_2	Ferredoxin-dependent glutamate synthase 1	
CAH1212707	xylB_2	Xylulose kinase	
CAH1216815	xylB_3		
CAH1218352	ackA	Acetate kinase	
CAH1218755	citB	Aconitate/2-methylaconitate hydratase	
CAH1221783	ldh1	L-lactate dehydrogenase 1	
CAH1222541	citZ	Citrate synthase 2	
CAH1222546	icd	Isocitrate dehydrogenase [NADP]	
CAH1222550	mdh	Malate dehydrogenase	
CAH1223443	kdgA_3		
CAH1224998	pflB	Formate acetyltransferase	
CAH1225003	pflA	Pyruvate formate-lyase 1-activating enzyme	

Table S9. Biosynthetic gene clusters of strain JJ-246ᵀ for secondary metabolites

accession	gene	annotated product	type
CAH1195583	-	-	Non-ribosomal peptide synthetase
CAH1195588	-	-	
CAH1195593	-	-	
CAH1195596	-	-	Type I polyketide synthase
CAH1195600	degU_2	Transcriptional regulatory protein	
CAH1195605	-	-	
CAH1195610	-	-	Trans-AT polyketide synthase
CAH1195615	virS	HTH-type transcriptional regulator	
CAH1195620	-	-	
CAH1195626	thrC	Threonine synthase	
CAH1195632	-	-	
CAH1195638	alkA	DNA-3-methyladenine glycosylase	
CAH1195644	-	-	
CAH1195650	-	-	
CAH1195656	-	-	
CAH1195662	yusV_2	putative siderophore transport system ATP-binding protein	
CAH1195668	zupT	Zinc transporter	
CAH1195673	dltA_1	D-alanine--D-alanyl carrier protein ligase	
CAH1195678	srfAA_1	Surfactin synthase subunit 1	
CAH1195685	-	-	
CAH1195691	pksM	Polyketide synthase	
CAH1195697	fenF	Malonyl CoA-acyl carrier protein transacylase	
CAH1195704	tycB_1	Tyrocidine synthase 2	
CAH1195710	dap_2	D-aminopeptidase	
CAH1195716	srfAA_2		
CAH1195722	-	-	
CAH1195728	-	-	
CAH1195733	-	-	
CAH1195741	greA_1	Transcription elongation factor	
CAH1195747	-	-	
CAH1195753	aziB	5-methyl-1-naphthoate synthase	
CAH1195760	-	-	
CAH1195766	-	-	
CAH1195772	-	-	
CAH1195778	-	-	
CAH1195784	-	-	
CAH1195790	-	-	
CAH1195797	-	-	
CAH1195804	-	-	
CAH1195811	-	-	
CAH1195817	yndE_1	Spore germination protein	
CAH1195823	gerAC_1	Spore germination protein A3	
CAH1195829	gerBA_1	Spore germination protein B1	
CAH1195835	-	-	
CAH1195841	-	-	
CAH1197609	yfhA_2	putative siderophore transport system permease protein	IucA/IucC-like siderophores
CAH1197611	yfiZ_2	putative siderophore transport system permease protein	Note: 25% similarity to BGC0000943, i.e. staphyloferrin B
CAH1197613	yfiY_3	putative siderophore-binding lipoprotein	
CAH1197615	ssuE_1	FMN reductase (NADPH)	
CAH1197617	sbnH	2-[(L-alanin-3-ylcarbamoyl)methyl]-2-hydroxybutanedioate decarboxylase	
CAH1197619	sbnE	L-2,3-diaminopropanoate--citrate ligase	
CAH1197621	sbnB_1	N-((2S)-2-amino-2-carboxyethyl)-L-glutamate dehydrogenase	
CAH1197623	sbnA_2	N-(2-amino-2-carboxyethyl)-L-glutamate synthase	
CAH1197625	sbnF_1	2-[(L-alanin-3-ylcarbamoyl)methyl]-3-(2-aminoethylcarbamoyl)-2-hydroxypropanoate synthase	
CAH1197627	sbnF_2		
CAH1197629	ntaA_1	Nitrilotriacetate monooxygenase component A	
CAH1197631	rhaS_2	HTH-type transcriptional activator	
CAH1197633	copZ_1	Copper chaperone	
CAH1197635	-	-	

Table S9. (cont.)

accession	gene	annotated product	type
CAH1200612	baeB	putative polyketide biosynthesis zinc-dependent hydrolase	agrD-like cyclic lactone autoinducer peptides
CAH1200613	bcrA_2	Bacitracin transport ATP-binding protein	
CAH1200614	-	-	
CAH1200615	rcsC_11	Sensor histidine kinase	
CAH1200616	-	-	
CAH1200617	-	-	
CAH1200618	-	-	
CAH1200619	agrB_1	Accessory gene regulator protein	
CAH1200620	-	-	
CAH1200622	-	-	
CAH1200624	-	-	
CAH1200626	-	-	
CAH1200628	-	-	
CAH1200630	-	-	
CAH1200632	-	-	
CAH1200634	-	-	
CAH1200636	-	-	
CAH1200639	-	-	
CAH1200642	bacC	Dihydroanticapsin 7-dehydrogenase	
CAH1200645	-	-	
CAH1200747	-	-	Non-ribosomal peptide synthetase
CAH1200750	-	-	
CAH1200753	-	-	
CAH1200756	-	-	
CAH1200759	melC_1	Melibiose/raffinose/stachyose import permease protein	
CAH1200762	lacF_4	Lactose transport system permease protein	
CAH1200765	-	-	
CAH1200768	-	-	
CAH1200771	-	putative response regulatory protein	
CAH1200773	-	-	
CAH1200775	-	-	
CAH1200777	-	-	
CAH1200779	-	-	
CAH1200781	-	-	
CAH1200783	-	-	
CAH1200785	dltA_2	D-alanine--D-alanyl carrier protein ligase	
CAH1200787	tycC_1	Tyrocidine synthase 3	
CAH1210909	-	-	Lasso peptide<br><br>Note: 80% similarity to BGC0001356 (i.e. paeninodin)
CAH1210913	-	-	
CAH1210917	-	-	
CAH1210920	btuD_19	Vitamin B12 import ATP-binding protein	
CAH1210923	-	-	
CAH1210926	-	-	
CAH1210929	-	-	
CAH1210933	-	-	
CAH1210936	-	-	
CAH1210940	-	-	
CAH1210944	-	-	
CAH1210948	-	-	
CAH1210952	-	-	
CAH1210956	-	-	
CAH1210960	-	-	
CAH1210964	tuaD_3	UDP-glucose 6-dehydrogenase	
CAH1210968	-	-	
CAH1210972	algA_1	Alginate biosynthesis protein	
CAH1210976	murB_1	UDP-N-acetylenolpyruvoylglucosamine reductase	
CAH1210980	wfgD	UDP-Glc:alpha-D-GlcNAc-diphosphoundecaprenol beta-1,3-glucosyltransferase	
CAH1210984	-	-	
CAH1210988	wbgU_2	UDP-N-acetylglucosamine 4-epimerase	

Table S9. (cont.)

accession	gene	annotated product	type
CAH1201441	qacR_1	HTH-type transcriptional regulator	Heterocyst glycolipid
CAH1201444	cpnA_1	Cyclopentanol dehydrogenase	synthase-like polyketide
CAH1201447	araR	Arabinose metabolism transcriptional repressor	synthase
CAH1201450	xylB_1	Xylulose kinase	
CAH1201453	araD	L-ribulose-5-phosphate 4-epimerase	
CAH1201456	araA	L-arabinose isomerase	
CAH1201459	-	-	
CAH1201461	-	-	
CAH1201464	-	-	
CAH1201467	-	-	
CAH1201470	nspC	Carboxynorspermidine/carboxyspermidine decarboxylase	
CAH1201473	-	Carboxynorspermidine synthase	
CAH1201476	-	-	
CAH1201479	-	-	
CAH1201482	-	-	
CAH1201485	-	-	
CAH1201488	-	-	
CAH1201491	-	-	
CAH1201494	-	-	
CAH1201497	acpP	Acyl carrier protein	
CAH1201500	hcaB	3-phenylpropionate-dihydrodiol/cinnamic acid-dihydrodiol dehydrogenase	
CAH1201503	ptlI_1	Pentalenene oxygenase	
CAH1201506	ptlI_2		
CAH1201509	-	-	
CAH1201510	-	-	
CAH1201513	-	-	
CAH1201516	wecE	dTDP-4-amino-4,6-dideoxygalactose transaminase	
CAH1201519	-	-	
CAH1201522	-	-	
CAH1201525	wecH	O-acetyltransferase	
CAH1201529	btuD_6	Vitamin B12 import ATP-binding protein	
CAH1201531	cysC_1	putative adenylyl-sulfate kinase	
CAH1206454	-	-	Non-ribosomal peptide
CAH1206547	-	-	synthetase cluster
CAH1206459	rhaS_6	HTH-type transcriptional activator	
CAH1206461	-	-	Note: 60% similarity to
CAH1206462	ytrA_1	HTH-type transcriptional repressor	BGC0000400 (i.e.
CAH1206464	-	-	paenibacterin)
CAH1206466	btuD_11	Vitamin B12 import ATP-binding protein	
CAH1206468	-	-	
CAH1206470	-	-	
CAH1206472	mdtD_2	Putative multidrug resistance protein	
CAH1206474	-	-	
CAH1206476	ephA	Epoxide hydrolase A	
CAH1206478	-	-	
CAH1206480	sigV_2	RNA polymerase sigma factor	
CAH1206482	-	-	
CAH1206484	-	putative FAD-linked oxidoreductase	
CAH1206486	hpd	4-hydroxyphenylpyruvate dioxygenase	
CAH1206488	ilvE	Branched-chain-amino-acid aminotransferase	
CAH1206490	srfAA_3	Surfactin synthase subunit 1	
CAH1206492	yfeW_2	Putative D-alanyl-D-alanine carboxypeptidase	
CAH1206495	lgrE_4	Linear gramicidin dehydrogenase	
CAH1206498	dltA_6	D-alanine--D-alanyl carrier protein ligase	
CAH1206501	grsB	Gramicidin S synthase 2	
CAH1206504	-	-	
CAH1206507	-	-	
CAH1206510	-	-	
CAH1206513	-	-	
CAH1206516	-	-	
CAH1206518	-	-	
CAH1206520	-	-	

Table S9. (cont.)

accession	gene	annotated product	type
CAH1202585	ureA	Urease subunit gamma	Non-ribosomal peptide synthetase<br><br>Note: 25 % similarity to BGC0000452 (i.e. tyrocidine)
CAH1202588	ureB1	Urease subunit beta 1	
CAH1202591	ureC	Urease subunit alpha	
CAH1202594	ureF	Urease accessory protein	
CAH1202597	ureG	Urease accessory protein	
CAH1202600	ureH	Urease accessory protein	
CAH1202603	-	-	
CAH1202606	-	-	
CAH1202609	-	-	
CAH1202612	-	-	
CAH1202615	-	-	
CAH1202618	yknX	Putative efflux system component	
CAH1202621	yknY_1	putative ABC transporter ATP-binding protein	
CAH1202624	macB	Macrolide export ATP-binding/permease protein	
CAH1202627	sigG_2	ECF RNA polymerase sigma factor	
CAH1202630	lgrE_1	Linear gramicidin dehydrogenase	
CAH1202633	tycC_2	Tyrocidine synthase 3	
CAH1202636	sbnA_3	N-(2-amino-2-carboxyethyl)-L-glutamate synthase	
CAH1202638	sbnB_2	N-((2S)-2-amino-2-carboxyethyl)-L-glutamate dehydrogenase	
CAH1202640	dltA_3	D-alanine--D-alanyl carrier protein ligase	
CAH1202642	ppsB	Plipastatin synthase subunit B	
CAH1202644	tycB_2	Tyrocidine synthase 2	
CAH1202646	tycC_3		
CAH1202648	msbA_1	Lipid A export ATP-binding/permease protein	
CAH1202650	-	Putative multidrug export ATP-binding/permease protein	
CAH1202652	-	-	
CAH1202654	ectB	Diaminobutyrate--2-oxoglutarate transaminase	
CAH1202656	pknD_3	Serine/threonine-protein kinase	
CAH1202658	prkC_1	Serine/threonine-protein kinase	
CAH1202660	recD2	ATP-dependent RecD-like DNA helicase	
CAH1202662	-	-	
CAH1202664	-	-	
CAH1202666	-	-	
CAH1202668	ytrE_1	ABC transporter ATP-binding protein	
CAH1202670	-	-	
CAH1202672	-	-	
CAH1202674	-	-	
CAH1202676	-	-	
CAH1212665	-	-	agrD-like cyclic lactone autoinducer peptides
CAH1212671	dinG_3	3'-5' exonuclease	
CAH1212677	-	Zinc-type alcohol dehydrogenase-like protein	
CAH1212682	COQ5_2	2-methoxy-6-polyprenyl-1,4-benzoquinol methylase	
CAH1212687	COQ5_3		
CAH1212692	cspB_2	Cold shock protein	
CAH1212697	yliI	Aldose sugar dehydrogenase	
CAH1212702	rcsC_17	Sensor histidine kinase	
CAH1212707	xylB_2	Xylulose kinase	
CAH1212712	-	-	
CAH1212717	-	-	
CAH1212721	agrB_2	Accessory gene regulator protein B	
CAH1212725	-	-	
CAH1212729	-	-	
CAH1212733	fabG_7	3-oxoacyl-[acyl-carrier-protein] reductase	
CAH1212737	-	putative HTH-type transcriptional regulator	
CAH1212741	-	-	

Table S9. (cont.)

accession	gene	annotated product	type
CAH1202706	-	-	Non-ribosomal peptide synthetase<br><br>Type I polyketide synthase<br><br>Note: 7% similarity to BGC0001059 (i.e. zwittermicin A)
CAH1202708	-	-	
CAH1202710	-	-	
CAH1202712	-	-	
CAH1202714	-	-	
CAH1202716	sigK_2	RNA polymerase sigma-K factor	
CAH1202718	cmpR_2	HTH-type transcriptional activator	
CAH1202720	tkt_2	Transketolase	
CAH1202722	-	-	
CAH1202724	menF_1	Isochorismate synthase	
CAH1202726	-	-	
CAH1202728	swrC_2	Swarming motility protein	
CAH1202730	lnrJ_1	Sensor histidine kinase	
CAH1202732	lnrK_7	Transcriptional regulatory protein	
CAH1202734	sbnA_4	N-(2-amino-2-carboxyethyl)-L-glutamate synthase	
CAH1202736	sbnB_3	N-((2S)-2-amino-2-carboxyethyl)-L-glutamate dehydrogenase	
CAH1202738	ppsD	Phthiocerol synthesis polyketide synthase type I	
CAH1202740	tycC_4	Tyrocidine synthase 3	
CAH1202742	lgrE_2	Linear gramicidin dehydrogenase	
CAH1202744	tycC_5	Tyrocidine synthase 3	
CAH1202746	mdrP	Na(+), Li(+), K(+)/H(+) antiporter	
CAH1202748	polC_2	DNA polymerase III PolC-type	
CAH1202750	-	-	
CAH1202752	-	-	
CAH1202754	mdtD_1	Putative multidrug resistance protein	
CAH1202756	ebrB	Multidrug resistance protein	
CAH1202758	ebrA	Multidrug resistance protein	
CAH1202760	betI_1	HTH-type transcriptional regulator	
CAH1202762	-	-	
CAH1202764	spoVAD	Stage V sporulation protein AD	
CAH1202766	-	-	
CAH1202767	-	-	
CAH1202769	-	-	
CAH1202771	-	-	
CAH1202773	-	-	
CAH1202775	yqeH	-	
CAH1202778	aroE	Shikimate dehydrogenase (NADP(+))	
CAH1202780	yhbY	RNA-binding protein	
CAH1202782	nadD	Nicotinate-nucleotide adenylyltransferase	
CAH1202784	-	-	
CAH1202786	rsfS	Ribosomal silencing factor	
CAH1202788	-	-	
CAH1218662	bamB_3	Outer membrane protein assembly factor	IucA/IucC-like siderophores<br><br>Note: 83% similarity to BGC0000942 (i.e. petrobactin), and 50% similarity to BGC0002683 (i.e. schizokinen) and BGC0002633 (i.e. schizokinen)
CAH1218668	dat	Diaminobutyrate--2-oxoglutarate aminotransferase	
CAH1218674	ddc	L-2,4-diaminobutyrate decarboxylase	
CAH1218680	iucA	N(2)-citryl-N(6)-acetyl-N(6)-hydroxylysine synthase	
CAH1218686	iucB	N(6)-hydroxylysine O-acetyltransferase	
CAH1218692	iucD	L-lysine N6-monooxygenase	
CAH1218697	-	-	
CAH1218706	sbnF_3	2-[(L-alanin-3-ylcarbamoyl)methyl]-3-(2-aminoethylcarbamoyl)-2-hydroxypropanoate synthase	
CAH1218711	menE	2-succinylbenzoate--CoA ligase	
CAH1218717	-	-	
CAH1218723	-	-	
CAH1218729	asbF	3-dehydroshikimate dehydratase	
CAH1218737	-	-	

Table S9. (cont.)

accession	gene	annotated product	type
CAH1205240	-	-	Non-ribosomal peptide synthetase
CAH1205246	-	-	
CAH1205253	-	-	
CAH1205260	khtT_1	K(+)/H(+) antiporter subunit	
CAH1205266	khtU_1	K(+)/H(+) antiporter subunit	
CAH1205272	emrB_2	Colistin resistance protein	
CAH1205278	-	-	
CAH1205286	-	-	
CAH1205293	khtT_2	K(+)/H(+) antiporter subunit	
CAH1205296	cntE	Staphylopine export protein	
CAH1205303	slyA_2	Transcriptional regulator	
CAH1205312	-	-	
CAH1205319	-	-	
CAH1205326	map_1	Methionine aminopeptidase 1	
CAH1205332	-	-	
CAH1205341	-	-	
CAH1205347	-	3'3'-cGAMP-specific phosphodiesterase 2	
CAH1205352	xerC_2	Tyrosine recombinase	
CAH1205357	iolG_4	Myo-inositol 2-dehydrogenase	
CAH1205361	-	-	
CAH1205365	yciC_1	Putative metal chaperone	
CAH1205368	dltA_4	D-alanine--D-alanyl carrier protein ligase	
CAH1205372	-	-	
CAH1205377	tycC_6	Tyrocidine synthase 3	
CAH1205381	lgrE_3	Linear gramicidin dehydrogenase	
CAH1205385	yojI	ABC transporter ATP-binding/permease protein	
CAH1205389	sfp_2	4'-phosphopantetheinyl transferase	
CAH1205393	dltA_5	D-alanine--D-alanyl carrier protein ligase	
CAH1205397	-	-	
CAH1205401	tycC_7	Tyrocidine synthase 3	
CAH1205405	kmo	Kynurenine 3-monooxygenase	
CAH1205409	-	-	
CAH1205413	-	-	
CAH1205417	-	-	
CAH1205421	-	-	
CAH1205426	-	-	
CAH1205431	-	-	
CAH1205436	-	-	
CAH1205442	-	-	
CAH1205447	-	-	
CAH1205452	gltC_2	HTH-type transcriptional regulator	
CAH1205457	-	-	
CAH1205463	-	-	
CAH1205468	potA	Spermidine/putrescine import ATP-binding protein	
CAH1205473	-	-	
CAH1225085	-	-	agrD-like cyclic lactone autoinducer peptides
CAH1225090	-	-	
CAH1225095	-	-	
CAH1225100	-	-	
CAH1225106	-	-	
CAH1225112	-	-	
CAH1225117	agrB_3	Accessory gene regulator protein B	
CAH1225122	-	-	
CAH1225127	-	-	
CAH1225132	kptA	putative RNA 2'-phosphotransferase	
CAH1225137	isp_1	Intracellular serine protease	
CAH1225143	isp_2		
CAH1225147	-	-	
CAH1225153	-	-	
CAH1225159	-	-	
CAH1225165	-	-	
CAH1225172	-	-	
CAH1225180	-	-	
CAH1225187	-	-	
CAH1225194	-	-	
CAH1225201	-	-	

Table S9. (cont.)

accession	gene	annotated product	type
CAH1208876	ilvD_2	Dihydroxy-acid dehydratase	Type III polyketide synthase
CAH1208881	rspR_1	HTH-type transcriptional repressor	
CAH1208884	-	putative HTH-type transcriptional regulator	
CAH1208889	-	-	Terpene
CAH1208893	-	-	
CAH1208897	-	-	Non-ribosomal peptide synthetase
CAH1208901	-	-	
CAH1208905	gerBA_6	Spore germination protein B1	
CAH1208909	gerBC_4	Spore germination protein B3	
CAH1208913	yndE_5	Spore germination protein	
CAH1208917	-	-	
CAH1208921	-	-	
CAH1208925	-	-	
CAH1208929	oxdD	Oxalate decarboxylase	
CAH1208933	hisC_2	Histidinol-phosphate aminotransferase	
CAH1208937	-	-	
CAH1208941	-	Heme uptake protein MmpL11	
CAH1208945	-	-	
CAH1208949	-	-	
CAH1208953	-	Alpha-pyrone synthesis polyketide synthase-like Pks18	
CAH1208957	-	-	
CAH1208961	mshA_6	D-inositol-3-phosphate glycosyltransferase	
CAH1208963	bmr3_3	Multidrug resistance protein 3	
CAH1208967	-	-	
CAH1208973	-	-	
CAH1208977	-	-	
CAH1208979	-	Alpha-amylase	
CAH1208983	purR_4	HTH-type transcriptional repressor	
CAH1208990	-	-	
CAH1208993	-	-	
CAH1208997	-	Peptidoglycan-N-acetylglucosamine deacetylase	
CAH1209001	-	-	
CAH1209005	-	-	
CAH1209009	gerN	Na(+)/H(+)-K(+) antiporter	
CAH1209012	-	-	
CAH1209016	noc_1	Nucleoid occlusion protein	
CAH1209020	-	-	
CAH1209024	gerBC_5	Spore germination protein B3	
CAH1209028	yndE_6		
CAH1209032	gerBA_7	Spore germination protein B1	
CAH1209036	-	-	
CAH1209041	-	-	
CAH1209045	hmp	Flavohemoprotein	
CAH1209049	dltA_7	D-alanine--D-alanyl carrier protein ligase	
CAH1209053	tycC_8	Tyrocidine synthase 3	
CAH1209056	-	-	
CAH1209059	vgb_1	Virginiamycin B lyase	
CAH1209062	-	-	
CAH1209065	-	-	
CAH1209068	-	-	
CAH1209072	-	-	
CAH1209076	-	IS4 family transposase ISDre1	
CAH1209080	-	-	
CAH1209084	rhtB	Homoserine/homoserine lactone efflux protein	
CAH1209091	-	-	
CAH1209095	-	-	
CAH1209099	-	-	
CAH1209102	ricR	Copper-sensing transcriptional repressor	
CAH1209106	dsrE2	Sulfur carrier protein DsrE2	
CAH1209110	-	-	
CAH1209114	gloB_2	Hydroxyacylglutathione hydrolase	
CAH1209118	glpE_2	Thiosulfate sulfurtransferase	
CAH1209122	-	-	
CAH1209126	trxB_2	Thioredoxin reductase	
CAH1209130	-	-	
CAH1209134	-	-	
CAH1209138	-	-	

Table S9. (cont.)

accession	gene	annotated product	type
CAH1215340	yfnB	Putative HAD-hydrolase	Non-ribosomal peptide synthetase
CAH1215343	yddE	putative isomerase	
CAH1215346	pphA	Serine/threonine-protein phosphatase 1	
CAH1215349	-	-	
CAH1215351	mrdB	Peptidoglycan glycosyltransferase	
CAH1215354	-	-	
CAH1215357	queE	7-carboxy-7-deazaguanine synthase	
CAH1215360	queC	7-cyano-7-deazaguanine synthase	
CAH1215363	-	-	
CAH1215366	mshA_8	D-inositol-3-phosphate glycosyltransferase	
CAH1215369	-	-	
CAH1215372	-	-	
CAH1215375	-	-	
CAH1215378	-	-	
CAH1215381	xpt	Xanthine phosphoribosyltransferase	
CAH1215384	-	-	
CAH1215388	-	-	
CAH1215391	-	-	
CAH1215394	-	-	
CAH1215397	dltA_8	D-alanine--D-alanyl carrier protein ligase	
CAH1215400	-	-	
CAH1215403	hprK_1	HPr kinase/phosphorylase	
CAH1215405	vgb_2	Virginiamycin B lyase	
CAH1215407	-	-	
CAH1215409	degA_3	HTH-type transcriptional regulator	
CAH1215411	-	-	
CAH1215413	cheB_13	Protein-glutamate methylesterase/protein-glutamine glutaminase	
CAH1215415	yteP_3	putative multiple-sugar transport system permease	
CAH1215417	-	-	
CAH1215419	-	-	
CAH1215421	-	-	
CAH1215423	sacC	Levanase	
CAH1226093	-	-	Resorcinol
CAH1226101	-	-	

Table S10. Cellular fatty acid profiles of strain JJ-246^T and type strains of closely related species of the genus *Paenibacillus*.
Strains: 1, JJ-246^T; 2, *Paenibacillus oenotherae* DLE-12^T; 3, *Paenibacillus xanthinilyticus* 11N27^T. Data for taxa 2 and 3 from Kim et al. (2015a) and Kim et al. (2015b), respectively. The strains were cultured on R2A Agar for 2 days and cells from the third streak quadrant of the agar plates were used. Values are percentages of the total fatty acids; -, not detected.

**fatty acid**	**1**	**2**	**3**
iso-$C_{14:0}$	2.1	2.8	6.31
$C_{14:0}$	1.5	7.9	2.1
iso-$C_{15:0}$	5.4	1.5	4.0
anteiso-$C_{15:0}$	45.6	45.4	45.3
$C_{15:0}$	3.5	-	-
iso-$C_{16:0}$	12.0	11.0	21.2
$C_{16:1}\omega 11c$	-	-	-
$C_{16:0}$	16.1	25.1	12.9
iso-$C_{17:0}$	5.8	TR	1.6
anteiso-$C_{17:0}$	5.1	3.4	4.1
$C_{18:1}\omega 9c$	-	1.4	-

Figure S1. Maximum-parsimony tree showing the phylogenetic position of strain JJ-246ᵀ among closest related *Paenibacillus* species. The tree was generated in ARB based on 16S rRNA gene sequences between positions 95 to 1446 according to *E. coli* numbering. GenBank accession numbers are given in parentheses. Numbers at branch nodes refer to bootstrap values >70% (100 replicates). Circle marks nodes that were also present in the maximum-likelihood tree. Larger circles were supported by high bootstrap values in the maximum-likelihood tree. Type strains of *Cohnella* species were used as outgroup. Bar, 0.1 substitutions per nucleotide position.

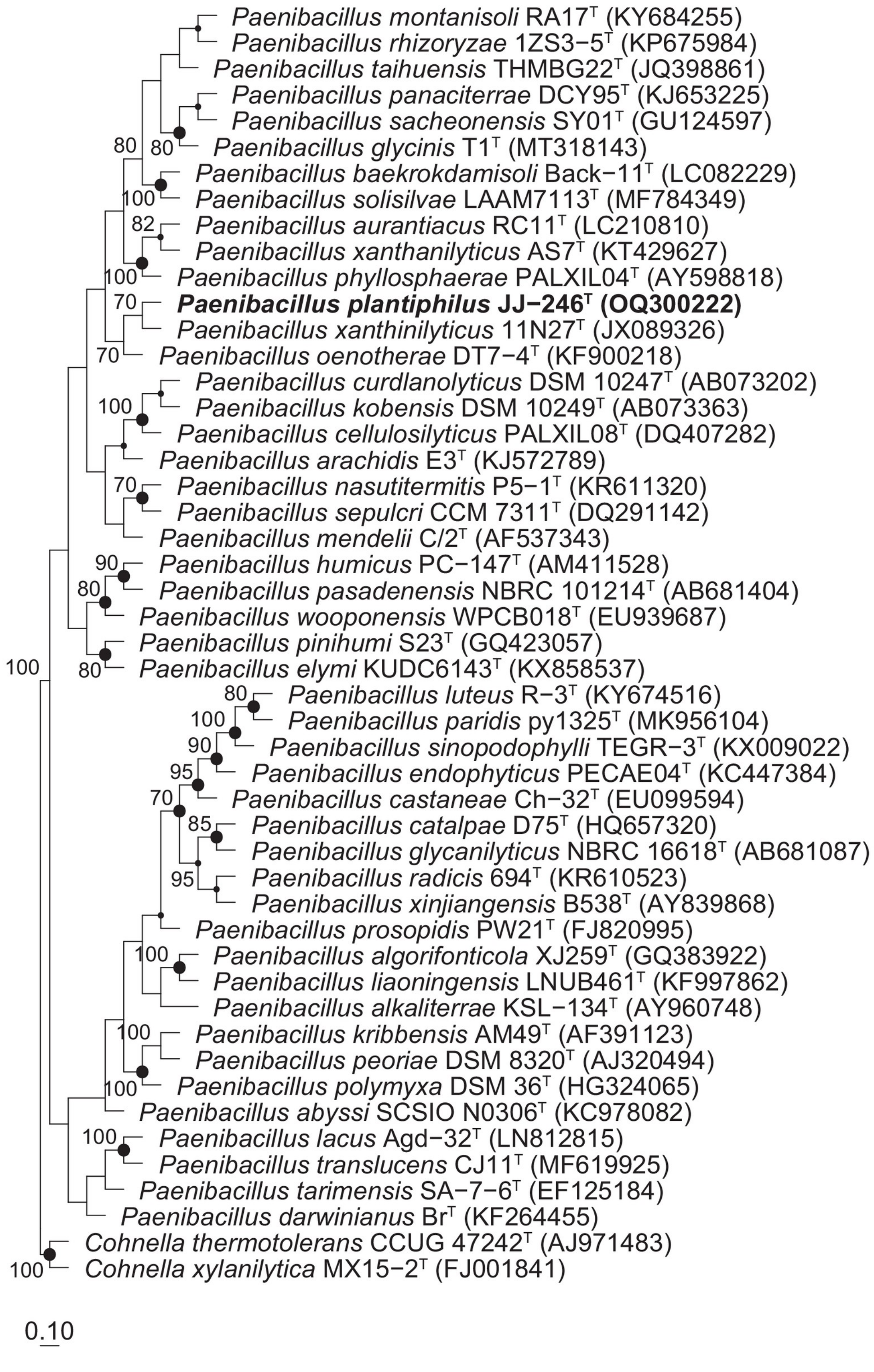

Figure S2. Phylogenetic tree showing the phylogenetic placement of strain JJ-246^T among type strains of closely related *Paenibacillus* species. This maximum likelihood tree was inferred from the concatenation of 120 multiple amino acid sequence alignments. Two *Cohnella* type strains were used as outgroup. The genome sequence accession is specified between parentheses next after each taxon name. Every internal branch is 100% supported by bootstrap analysis (500 replicates). Bar, 0.05 amino acid substitutions per site.

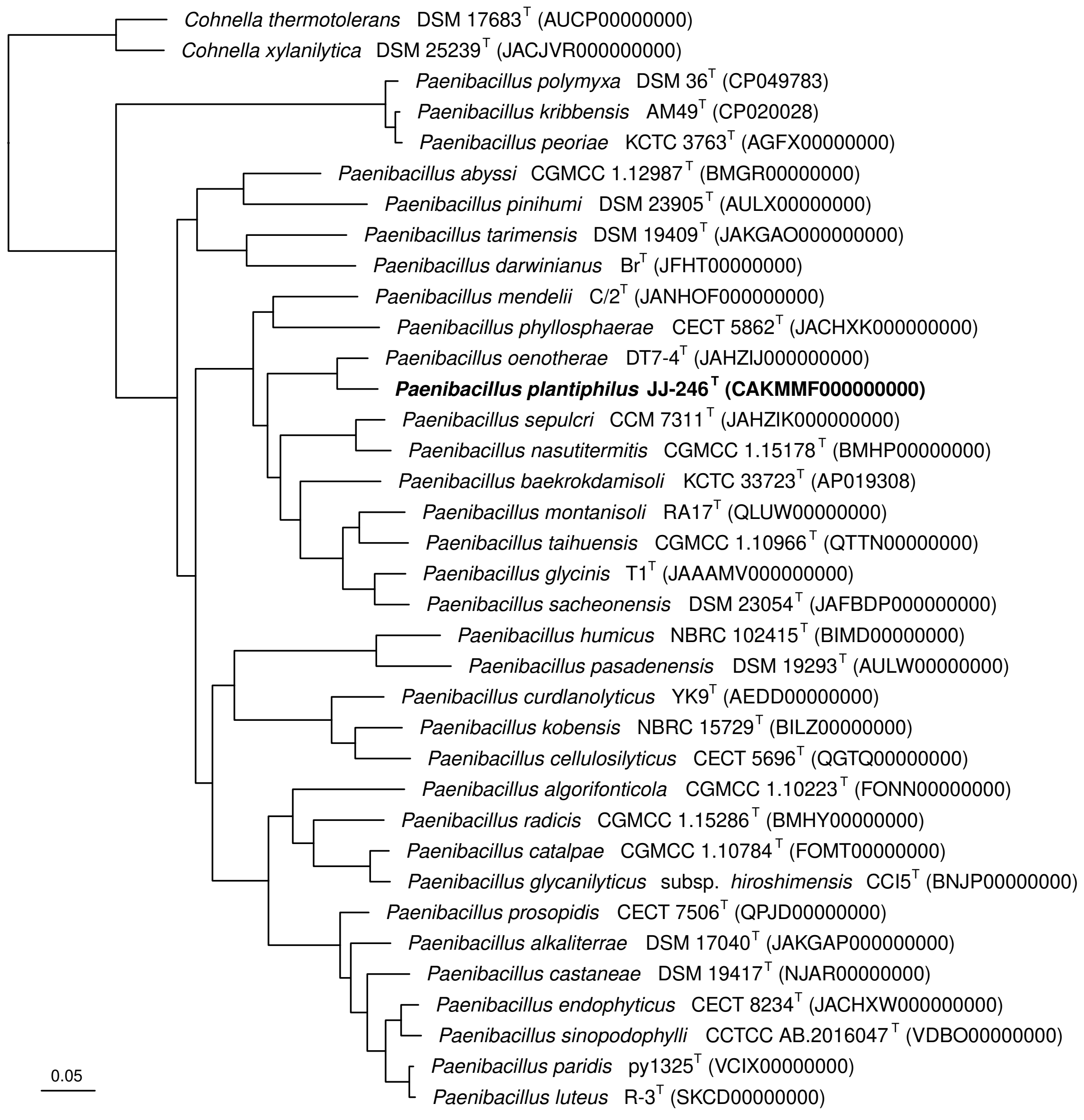

Figure S3. Polar lipid profile of strain JJ-246^{T} after successive staining of a chromatographic plate with ninhydrin (aminolipids), molybdenum blue reagent (phospholipids) and sulfuric acid with heating at 140°C (all lipids).
Appreviations: PG, phosphatidylglycerol; DPG, diphosphatidylglycerol; PE, phosphatidylethanolamine; PS, phosphatidylserine; APL, one unidentified aminophospholipid.

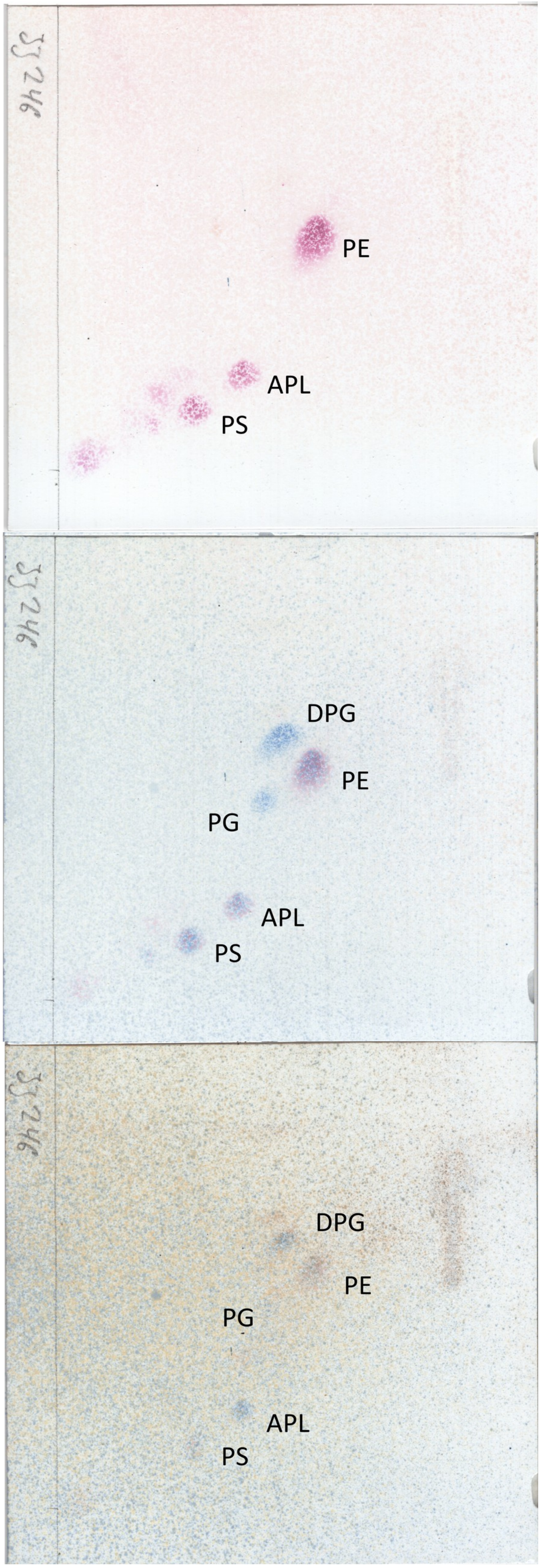

Text S1. Bioinformatic command line summary

Phylogenetic classification using JolyTree v2.1
URL: https://gitlab.pasteur.fr/GIPhy/JolyTree
Input: a directory `genomes/` containing 36 FASTA-formatted genome assemblies
Output: a NEWICK file `paenibacillus.nwk` containing the genome-based phylogenetic tree

```
JolyTree.sh -i genomes -b paenibacillus -s 0.8 -k 27 -t 95
```

Coding sequence extraction using eCDS v1.1
URL: https://gitlab.pasteur.fr/GIPhy/eCDS
Input: a SMP file `gene.smp` containing a reference position-specific scoring matrix associated to a given gene and a FASTA-formatted genome assembly file `genome.fa`
Output: a FASTA file `cds.faa` containing the translated homologous coding sequence extracted from the specified genome

```
eCDS.sh -q gene.smp -s genome.fa -c 30 -p 30 -f -S 2 -o cds
```

Multiple amino acid sequence alignment building using MAFFT v7.467
URL: https://mafft.cbrc.jp/alignment/software
Input: a FASTA file `seq.fa` containing homologous ammino acid sequences
Output: a FASTA file `msa.fa` containing the multiple sequence alignment

```
mafft --globalpair --maxiterate 1000 --allowshift --unalignlevel 0.7 seq.fa > msa.fa
```

Selection of aligned characters suited for phylogenetic analysis using BMGE v2.0
URL: https://research.pasteur.fr/en/software/bmge-block-mapping-and-gathering-with-entropy
Input: a FASTA file `msa.faa` containing a multiple amino acid sequence alignment
Output: a FASTA file `msa.ffa` containing the filtered multiple sequence alignment

```
BMGE -i msa.faa -t AA -o msa.ffa
```

Concatenation of the 120 filtered multiple sequence alignments using Concatenate v1.1b
URL: https://gitlab.pasteur.fr/GIPhy/Concatenate
Input: a text file `datafiles.txt` containing the list of the FASTA-formatted alignments to concatenate
Output: a FASTA file `sm.fa` containing the concatenated multiple sequence alignments

```
Concatenate -i datafiles.txt -f -o sm.fa
```

Phylogenetic tree inference using IQ-TREE v2.2.2.2
URL: http://www.iqtree.org
Input: a FASTA file `sm.fa` containing the concatenation of 120 multiple sequence alignments
Output: a NEWICK file `sm.fa.treefile` containing a maximum likelihood tree

```
iqtree2 -s sm.fa --cmax 20 -b 500 -T 12
```
